# A case of perihilar cholangiocarcinoma with peritoneal dissemination that achieved a pathological complete response using immune checkpoint inhibitors: A case report

**DOI:** 10.1007/s13691-025-00842-2

**Published:** 2026-01-12

**Authors:** Takehiko Saijo, Hideaki Sato, Shuichi Aoki, Masahiro Iseki, Mika Ando, Yuichiro Umino, Mitsuhiro Shimura, Koetsu Inoue, Daisuke Douchi, Takayuki Miura, Shimpei Maeda, Masaharu Ishida, Masamichi Mizuma, Kazuhiro Kikuta, Atsushi Masamune, Keigo Murakami, Toru Furukawa, Michiaki Unno

**Affiliations:** 1 Department of Surgery, Tohoku University Graduate School of Medicine, 1-1 Seiryo-Machi, Aoba-Ku, Sendai, 980-8574 Japan; 2 Division of Gastroenterology, Tohoku University Graduate School of Medicine, 1-1 Seiryo-Machi, Aoba-Ku, Sendai, 980-8574 Japan; 3 Department of Investigative Pathology, Tohoku University Graduate School of Medicine, 2-1 Seiryo-Machi, Aoba-Ku, Sendai, 980-8575 Japan

**Keywords:** Immune checkpoint inhibitor, Pathological complete response, Perihilar cholangiocarcinoma, Biliary tract cancer, Staging laparoscopy

## Abstract

Surgical resection is considered the only potentially curative treatment for biliary tract cancer (BTC). However, for patients with locally advanced or metastatic BTC, systemic chemotherapy remains the primary therapeutic option. Historically, chemotherapies such as gemcitabine plus cisplatin (GC), gemcitabine plus S-1, and GC plus S-1 have been employed; however, the prognosis remains poor. Recently, combined therapy with immune checkpoint inhibitors (ICIs) has emerged as a promising therapeutic approach for unresectable BTC. In cases with long-term efficacy to systemic chemotherapy, sequential surgical resection, known as conversion surgery, has also shown potential to improve overall outcomes in unresectable BTC. This case report presents a case of advanced perihilar cholangiocarcinoma with histologically confirmed peritoneal dissemination that achieved a pathological complete response after combination therapy with GC and durvalumab, followed by conversion surgery. Achieving a pathological complete response in the presence of peritoneal dissemination is rare. This case provides valuable insights into treatment strategies for this aggressive malignancy, demonstrating that a pathological complete response is possible even in the presence of peritoneal dissemination.

## Introduction

Biliary tract cancer (BTC) is a heterogeneous group of malignant tumors with an extremely poor prognosis [1, 2]. While surgical resection remains the only potentially curative treatment, the surgical procedure inherently involves a spectrum of risks, including postoperative complications and mortality [3, 4]. Patients with distant metastases or locally advanced BTC are generally considered unresectable, as achieving an R0 resection is often technically infeasible, and the presence of distant metastasis itself precludes curative intent.

For patients with unresectable BTC, the gemcitabine-based regimens are widely recognized as the first-line option [5–7]. Recent advancements have introduced immune checkpoint inhibitors (ICIs) as a pivotal approach in oncology. Clinical trials such as TOPAZ-1 and KEYNOTE-966 have shown potential benefits, providing hope for improved outcomes in this challenging disease, as well as other cancers [8–11].

Conversion surgery has emerged as a viable option for patients with initially unresectable BTC who respond favorably to systemic therapy. This approach involves preoperative treatment to achieve tumor downstaging, thereby enabling surgical resection [12–14]. These findings underscore the potential of conversion surgery to significantly improve long-term outcomes in selected patients. However, recurrence remains a major challenge even in resectable BTC cases, particularly in the presence of lymph node metastases or R1 resections [15, 16]. Achieving a pathological complete response (pCR) remains a rare event, particularly in highly advanced settings.

Here, we present a case of a patient with initially unresectable perihilar cholangiocarcinoma (PCCA) with peritoneal dissemination that achieved a pCR following combination therapy with gemcitabine, cisplatin, and durvalumab (GCD) and subsequently underwent conversion surgery. This case highlights the potential role of ICIs in achieving marked therapeutic responses and suggests a valuable role for staging laparoscopy in guiding treatment strategy for advanced BTC.

### Case presentation

**Fig. 1:**
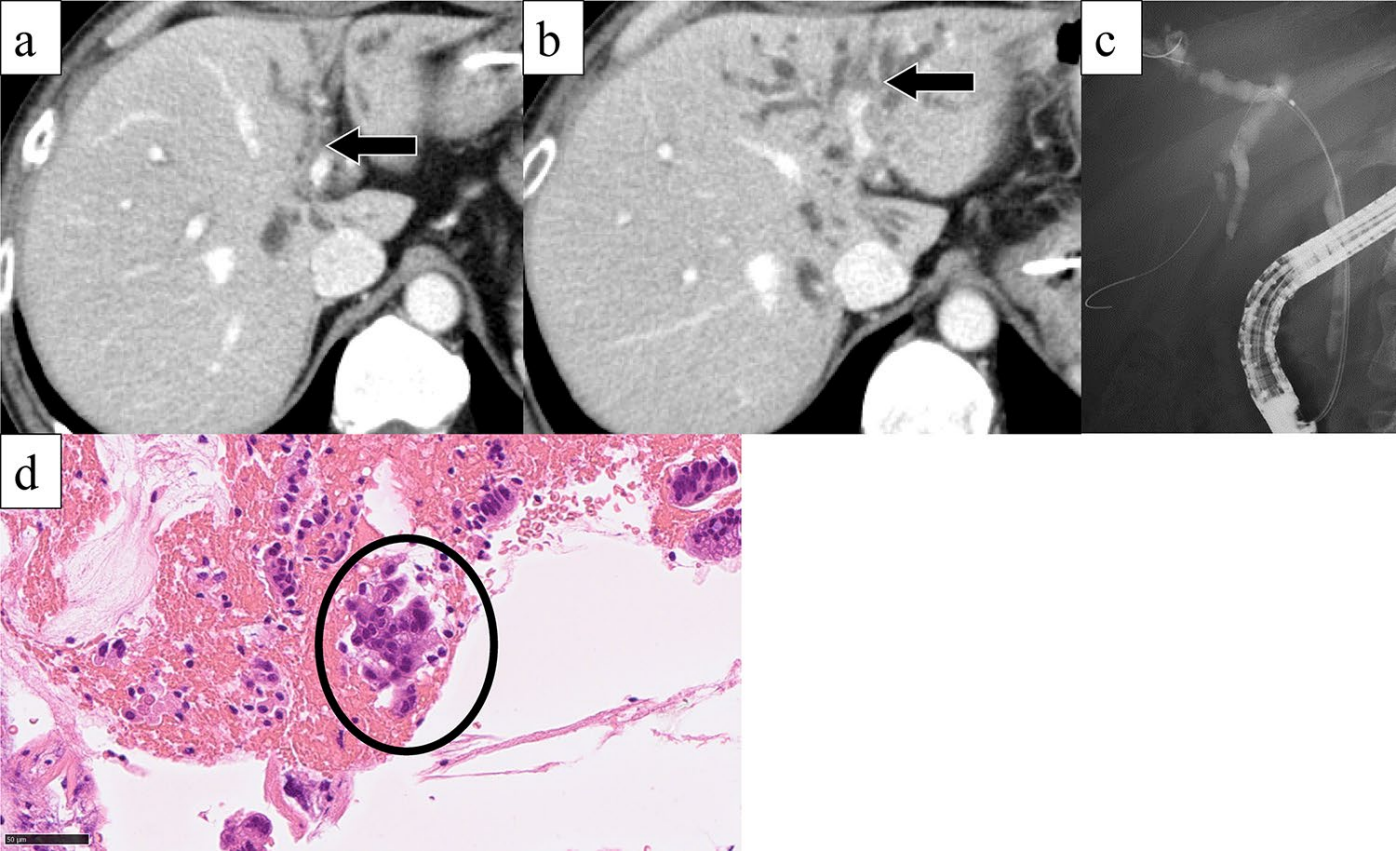
(**a**) Parietal enhancement is observed from the common bile duct to the left hepatic bile duct. (**b**) partial thrombosis of the left portal vein is present. (**c**): ERC classified the tumor as type IV according to the bismuth classification, and a biopsy confirmed adenocarcinoma. (**d**) the bile duct biopsy specimen shows fragmented atypical epithelium with nuclear enlargement, suggesting adenocarcinoma (hematoxylin–eosin staining)

**Fig. 2:**
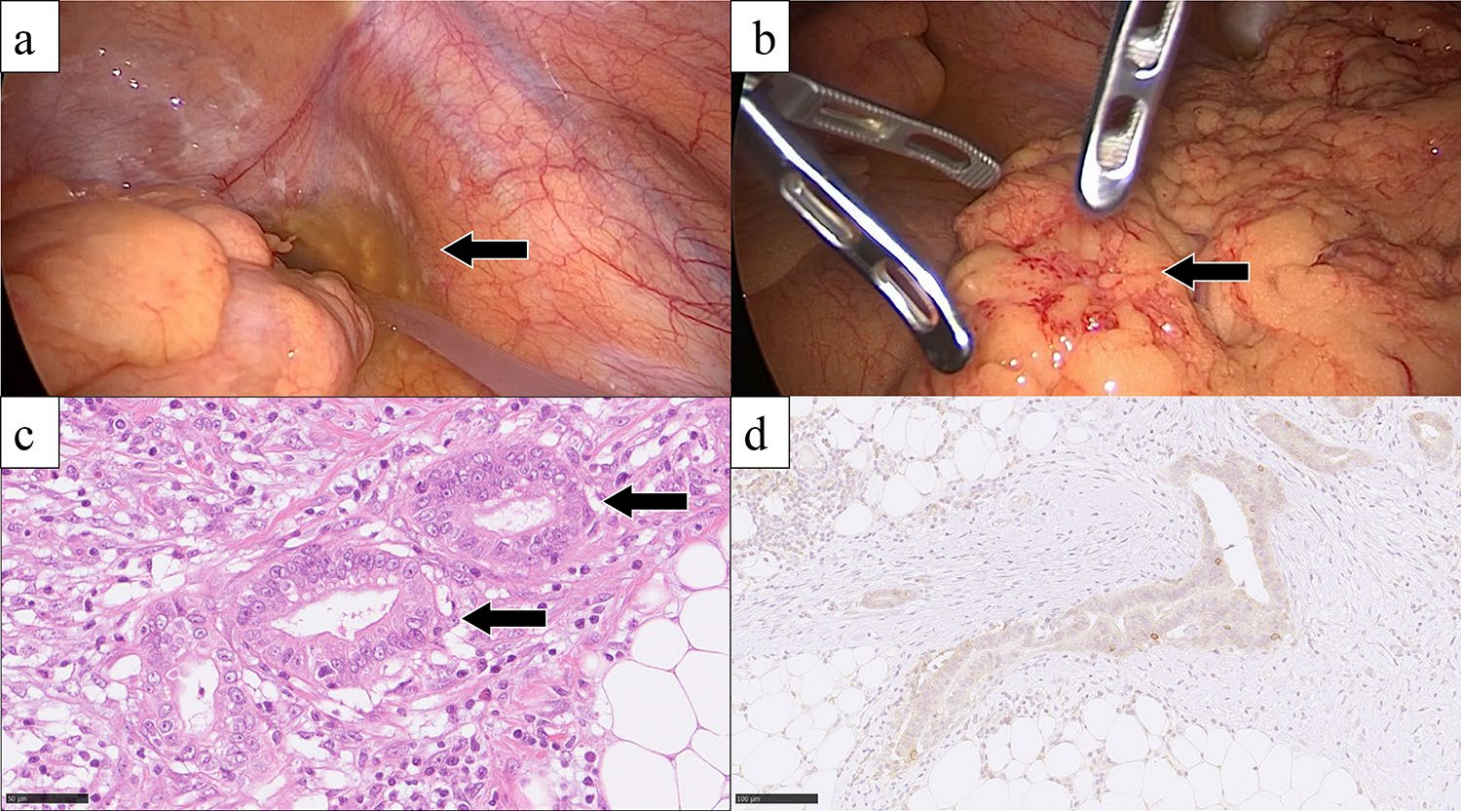
(**a**) Peritoneal nodules are present, and peritoneal cytology is positive. (**b**) the nodule found in the omentum was firm and partially accompanied by hemorrhage. (**c**) microscopic findings of the dissected omental node. the cells of omental node show nuclear enlargement forming glandular structures, consistent with adenocarcinoma. (hematoxylin–eosin staining) (**d**) the expression of PD-L1 was low (immunohistochemical staining)

**Fig. 3:**
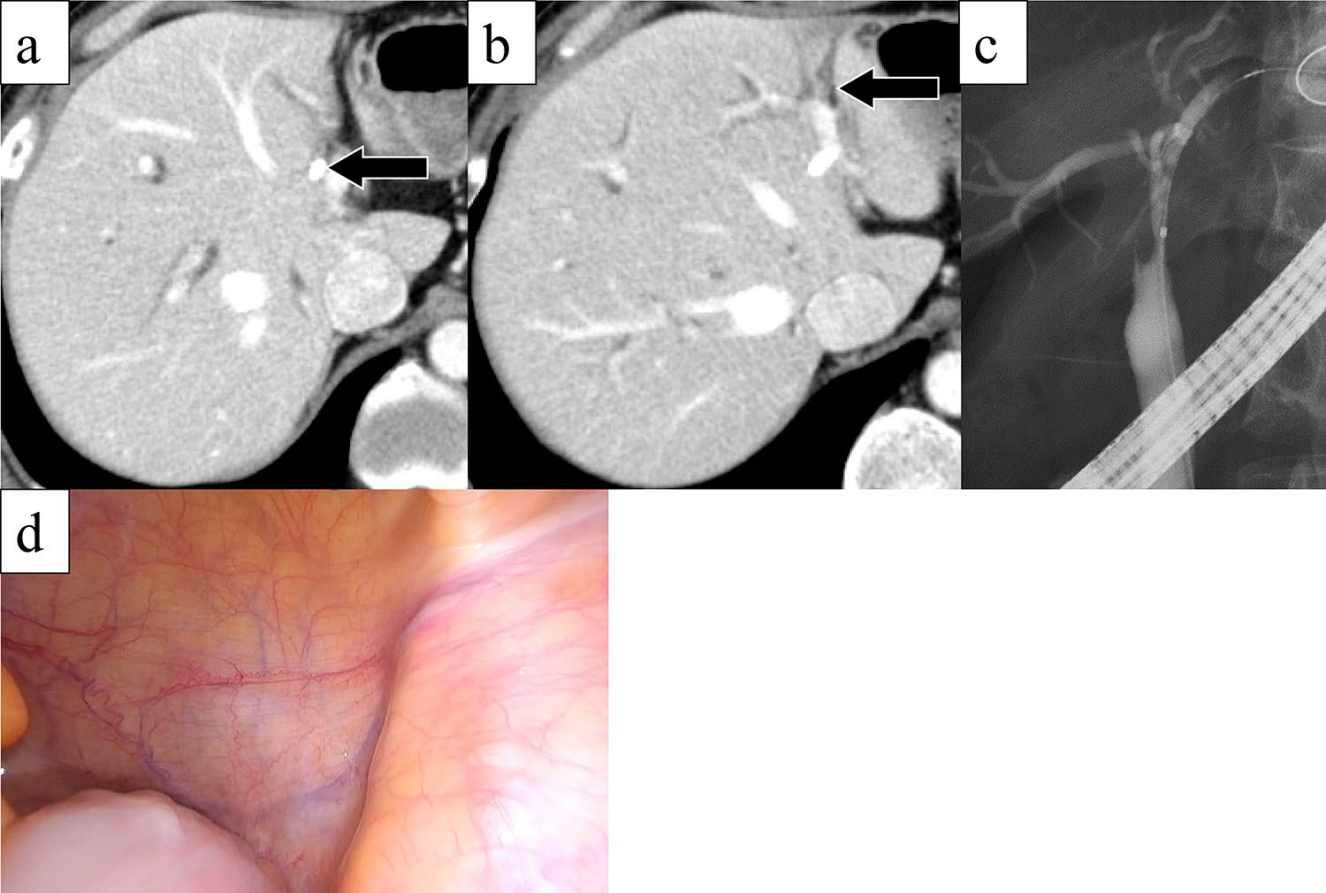
(**a**, **b**) The thickening of the bile duct wall and the dilation of the intrahepatic bile ducts have slightly improved. (**c**) after chemotherapy, ERC reclassified the tumor as type II according to the bismuth classification. (**d**) second staging laparoscopy revealed no evidence of peritoneal dissemination, and peritoneal cytology was negative

**Fig. 4:**
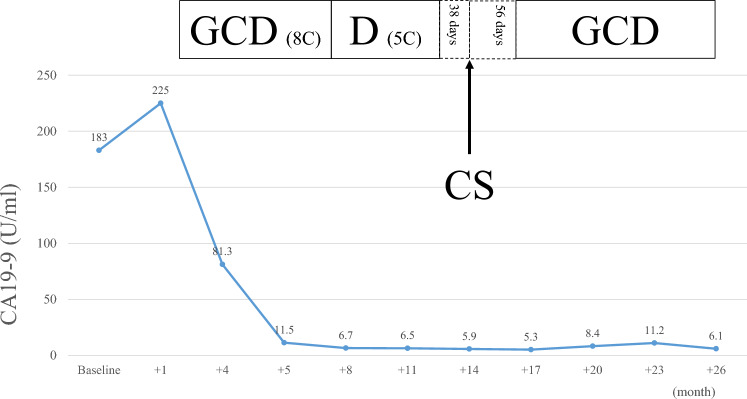
Changes in serum CA19-9 levels over the course of treatment. the patient’s serum CA19-9 level was 183 U/mL at the initial diagnosis. It rapidly decreased to 5.9 U/mL after 3 months of GCD therapy. the level remained consistently low throughout the preoperative treatment period until conversion surgery, and remained within the normal range postoperatively. Abbreviations; GCD, gemcitabine, cisplatin, and durvalumab; D, durvalumab; CS, conversion surgery

**Fig. 5:**
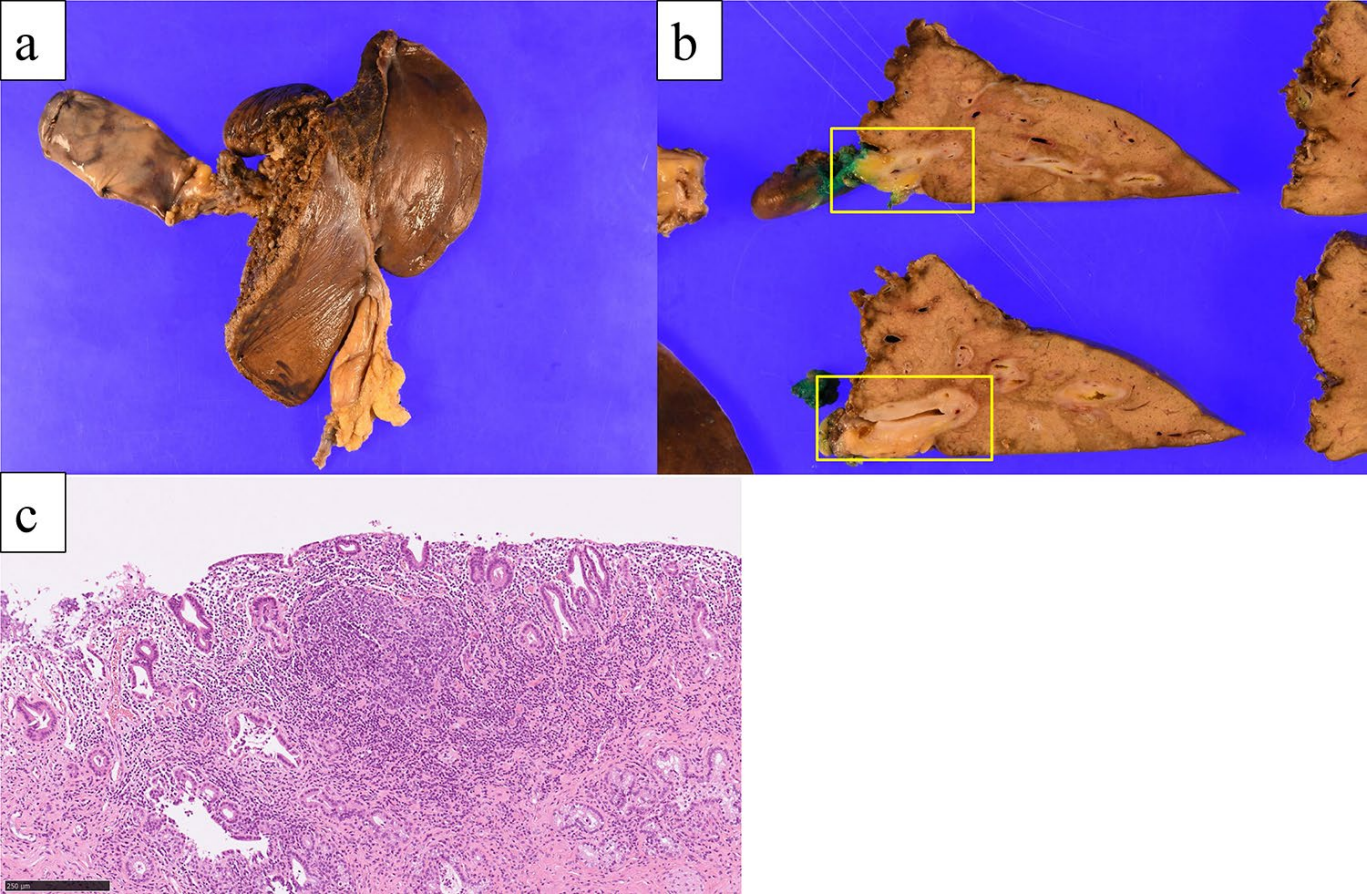
(**a**, **b**) In the macroscopic findings of the resected specimen, the bile duct wall is markedly thickened and exhibits a pale, whitish coloration. (**c**) in the microscopic findings of the resected specimen, fibrotic thickening of the bile duct wall was found with no residual malignant cells

A 50-year-old man presented with jaundice and right hypochondriac pain. A computed tomography (CT) scan revealed a 31 mm parietal enhancement extending from the common bile duct to the left hepatic bile duct, direct invasion into the right hepatic artery, bile duct dilation in the left lobe, partial thrombosis of the left portal vein, and lymphadenopathy in the hepatoduodenal ligament (Fig. 1a, b). Endoscopic retrograde cholangiography (ERC) classified the tumor as type IV according to the Bismuth classification (Fig. 1c), and a biopsy confirmed adenocarcinoma (Fig. 1d). Positron emission tomography with fluorodeoxyglucose (FDG-PET) demonstrated weak FDG uptake in the tumor, with no evidence of distant metastasis. Although preoperative imaging suggested a locally confined PCCA, staging laparoscopy was planned due to the appearance of relatively advanced disease. Staging laparoscopy revealed malignant peritoneal cytology, and a biopsy of an omental node confirmed adenocarcinoma (Fig. 2a-c). Immunohistochemical analysis of omental node showed weak PD-L1 expression (less than 1%) (Fig. 2d). Comprehensive genomic profiling revealed that microsatellite instability (MSI) was stable, and the tumor mutation burden (TMB) was low. Additionally, genetic alterations were identified in genes such as *ERBB3*, *TP53*, and *NF1*. Based on these findings, the patient was diagnosed with PCCA, classified as T3N1M1, Stage IVB according to the 8th edition of the UICC TNM classification [17]. The patient received a total of 8 cycles of GCD, followed by 5 cycles of durvalumab monotherapy. During the treatment, CT imaging demonstrated gradual tumor shrinkage, reducing to 21 mm (Fig. 3a, b), and ERC reclassified the tumor as type II according to the Bismuth classification (Fig. 3c). According to RECIST v1.1 criteria [18], the patient achieved a partial response (PR). CA 19–9 rapidly decreased from 183 U/mL to 5.9 U/mL, reaching normal levels, in three months from initial treatment and remained within the normal range for the subsequent nine months (Fig. 4). The second staging laparoscopy revealed no evidence of peritoneal dissemination, and both the peritoneal cytology and the biopsy of an omental node were pathologically negative (Fig. 3d). Based on these findings, conversion surgery was planned 51 weeks after the initial treatment. The patient underwent a left hepatectomy with caudate lobectomy, combined with resection of the extrahepatic bile duct and wedge resection of the portal vein. The right hepatic artery, which had been suspected of tumor invasion prechemotherapy, exhibited severe inflammatory changes during surgery. However, no macroscopic evidence of tumor infiltration was observed, and the artery was successfully dissected without the need for combined resection. The postoperative course was uneventful, and he was discharged 20 days after surgery. Pathological examination of the resected specimen revealed fibrotic thickening of the perihilar bile duct wall with extensive lymphocyte infiltration and no residual malignant cells, confirming a pCR (Fig. 5a-c). Postoperatively, GCD therapy was resumed, and the patient remains recurrence-free eleven months after the surgery.

## Discussion

Pathological complete response (pCR) is an exceedingly rare event in BTC, although several cases have been documented. For example, in ICC, Zhang et al. and Abudalou et al. reported cases of pCR in ICC following combination therapy with ICIs and standard chemotherapy regimens [19, 20]. Similarly, Wang et al. observed pCR in 2 of 13 patients with PCCA who underwent conversion surgery [21]. These findings highlight the potential of integrating ICIs with chemotherapy to achieve profound tumor responses in aggressive BTC cases.

In our case, pathological examination showed no residual malignant cells, indicating a pCR following chemotherapy combined with ICIs. This outcome is especially notable because the patient presented with peritoneal dissemination, which is recognized as a particularly adverse prognostic factor for unresectable cholangiocarcinoma [22]. A comprehensive PubMed search using the terms (“BTC” OR “cholangiocarcinoma”) AND “peritoneal dissemination” AND “complete response” yielded no prior reports of pCR in this setting. While other cases of pCR in BTC have been reported, as shown in Table 1 [19, 20, 23–25], our case is the first to achieve pCR in the presence of histologically confirmed peritoneal dissemination. This conclusion is supported by pathological clearance of malignancy in both the primary tumor and the biopsied omental lesion at the second staging laparoscopy. This exceptional response provides crucial insight into the potential of this treatment strategy for managing one of the most challenging presentations of BTC.

**Table 1:** Reported cases of pathological complete response (pCR) in biliary tract cancer. our case is the first reported case of pCR in a patient with peritoneal dissemination

Reference	Age (year)	Sex (M: F)	Stage	Chemotherapy	Adjuvant	Genetic alterations	Outcome (month)
20	36	M	IIIB	Gem+Cis+nabPTX+Camrelizumab	Gem+Cis+nabPTX+Camrelizumab		1
21	47	M		Gem+Cis+nab-PTX+Pembrolizumab	Pembrolizumab	TMB-H	6
25	64	M	IVB	Gem+Cis+Durvalumab		dMMR	10
26	69	M	IVA	Gem+Cis+Pembrolizumab		MSI-H, TMB-H	4
Our case	50	M	IVB	Gem+Cis+Durvalumab	Gem+Cis+Durvalumab	ERBB3, TP53, NF1	11

Abbreviations: gem, gemcitabine; Cis, cisplatin; nab-PTX, nab-paclitaxel

In the present case, an important aspect was the implementation of histopathological assessment by means of staging laparoscopy at critical junctures, which proved instrumental in determining the optimal timing of surgical intervention. Staging laparoscopy was undertaken both prior to and following treatment, thereby confirming the absence of peritoneal dissemination and demonstrating the subsequent complete response of peritoneal disease. Beyond its role in initial diagnosis, staging laparoscopy functioned as a critical therapeutic monitoring and decision-making tool. To the best of our knowledge, no comparable case has been previously documented. Given that positive peritoneal cytology has been reported to significantly correlate significantly with postoperative recurrence in locally advanced BTC [26], staging laparoscopy may be regarded as a highly valuable modality for guiding therapeutic strategies in this context. In advanced BTC, the presence of peritoneal micrometastasis often mandates systemic therapy. Our case highlights that staging laparoscopy can not only detect occult dissemination (pre-treatment) but also histologically confirm the complete eradication of the high-risk peritoneal lesions (post-treatment). This contrasts with imaging alone, which often cannot reliably exclude micrometastasis. Similar to its established role in evaluating response to neoadjuvant therapy in gastric or pancreatic cancer, staging laparoscopy in BTC can provide the definitive pathological evidence necessary to proceed with high-risk conversion surgery, thereby reducing the risk of futility and optimizing patient selection. Moreover, its capacity to procure tissue specimens offers the additional advantage of facilitating comprehensive genomic profiling. For these reasons, our institution actively employs staging laparoscopy for locally advanced BTC (such as cases requiring vascular resection or with extensive lymph node metastases), even in the absence of radiologically evident peritoneal metastases.

PD-L1 expression was not high in our case. While PD-L1 expression is often considered a prognostic factor, reported data are frequently inconsistent. The TOPAZ-1 trial demonstrated that durvalumab prolonged progression-free survival regardless of PD-L1 expression [8]. Conversely, some studies have shown that patients with low PD-L1 expression tend to exhibit better five-year and overall survival rates compared to those with high expression [27, 28]. Our case further supports this evidence, showing a marked therapeutic effect despite low PD-L1 expression. However, significant uncertainty remains regarding the relationship between PD-L1 expression and patient eligibility for conversion surgery or the achievement of pCR, thus emphasizing the critical need for further case accumulation.

Currently, no consensus exists regarding the optimal duration of preoperative therapy before conversion surgery in BTC [11]. However, the median duration of response (DOR) in patients treated with ICI combination therapy in the TOPAZ-1 and KEYNOTE-966 trials were approximately six months, which may serve as a relevant reference point for sustained disease control. In our institutions, conversion surgery was attempted to perform after achieving effective disease control for more than six months, supported by several favorable indicators, including the absence of peritoneal dissemination, no FDG uptake on PET, normalization of CA19-9 and a sustained response to therapy. Although some guidance can be drawn from pancreatic cancer and BTC treatment, in which sustained disease control for at least 6–8 months is often recommended [13, 29], the DOR data offers a more directly applicable benchmark for ICI-based conversion strategy in BTC. While DOR serves as an important measure of sustained control, the time to response (TTR) is also a critical factor. In our case, CA19-9 levels normalized rapidly within three months, followed by maintained radiologic and biochemical response. Our decision to proceed with surgery after a rapid biochemical response and confirmed disease clearance at 51 weeks aligns with the principle of ensuring both maximal tumor shrinkage/clearance (TTR) and sustained disease control (DOR) prior to attempting curative resection.

However, the question remains whether surgical intervention further improves long-term survival in patients achieving clinical complete response (cCR) with ICIs. In colorectal cancer, a watch-and-wait strategy is a viable option for patients with cCR after ICI treatment, particularly for those with deficient mismatch repair or MSI-high status [30]. This approach could also be a potential option for patients with BTC. However, while ICC allows for direct monitoring of the primary lesion, PCCA often presents challenges in primary tumor surveillance. This uncertainty underscores the need for additional clinical data and robust prospective studies. Furthermore, the management of pCR cases presents unique challenges, such as determining the role of adjuvant therapy, optimal surveillance strategies, long-term outcomes, recurrence rates, and quality of life. Addressing these issues is essential for establishing comprehensive management protocols. Although Capecitabine and S-1 are established standards for adjuvant therapy based on randomized controlled trials [31, 32], there is no clear evidence supporting adjuvant therapy following conversion surgery cases. In our patient, the decision was guided by high-risk factors (initial peritoneal dissemination) and the remarkable efficacy achieved with the preoperative GCD regimen, which resulted in pCR. We prioritized maintaining the therapeutic momentum by continuing the highly effective preoperative regimen, considering it a reasonable strategy for a high-risk patient.

In conclusion, we report a case of PCCA with peritoneal dissemination histologically confirmed pCR through a combination of ICIs and chemotherapy. This case highlights the potential role of ICIs and conversion surgery in BTC management, offering insights into therapeutic strategies for this aggressive disease. Future efforts should focus on identifying reliable biomarkers to predict which patients are most likely to benefit from conversion surgery, thereby optimizing patient selection and improving outcomes.

## Data Availability

Not applicable.
